# Supplementary figures and images for: Assessing racial bias in type 2 diabetes risk prediction algorithms
Source: PLOS Glob Public Health. 2023 May 17;3(5):e0001556. doi: 10.1371/journal.pgph.0001556 (PMC10191313; doi:10.1371/journal.pgph.0001556)

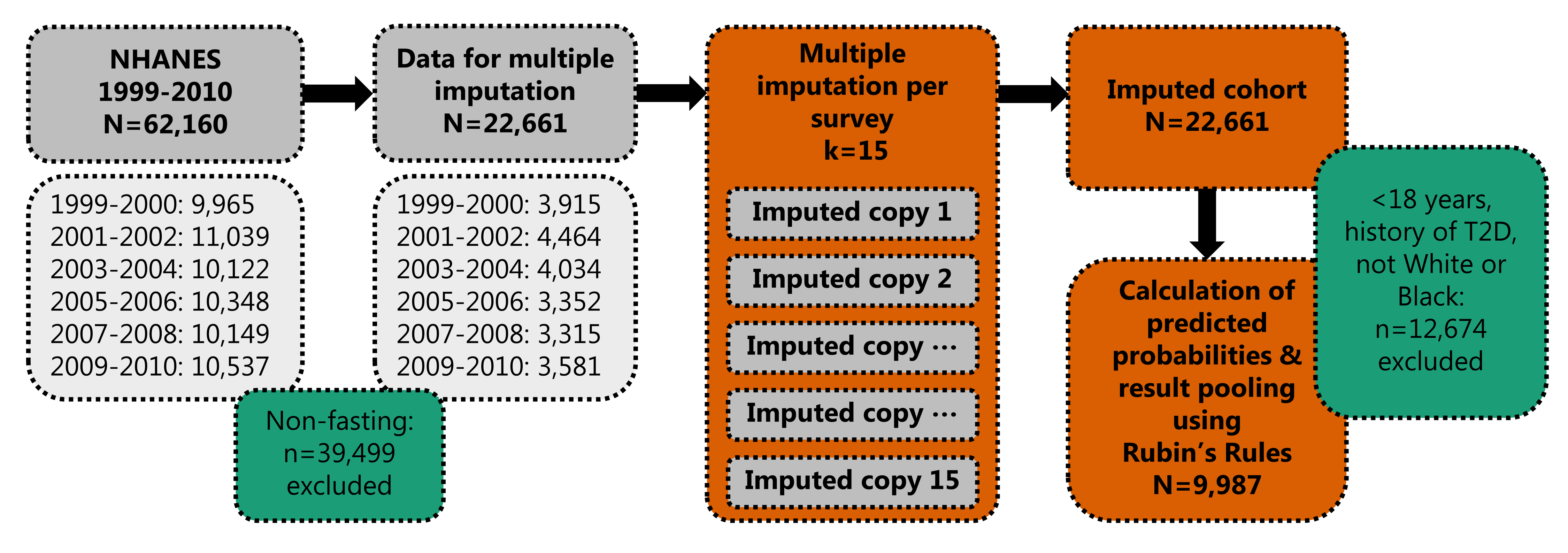

Supplement: S1 Fig — (TIF) [file pgph.0001556.s001.tif]
