# Supplementary material for: Assessing racial bias in type 2 diabetes risk prediction algorithms
Source: PLOS Glob Public Health. 2023 May 17;3(5):e0001556. doi: 10.1371/journal.pgph.0001556 (PMC10191313; doi:10.1371/journal.pgph.0001556)
Supplement: Attachment — Submitted filename: Response_to_Reviewers.docx [file pgph.0001556.s006.docx]

Response to Reviewers

*Assessing racial bias in type 2 diabetes risk prediction algorithms*

*PGPH-D-23-00034*

**Editor:**

E.1: Thank you for submitting your manuscript to PLOS Global Public Health. After careful consideration, we feel that it has merit but does not fully meet PLOS Global Public Health’s publication criteria as it currently stands. Therefore, we invite you to submit a revised version of the manuscript that addresses the points raised during the review process.

We have noticed that you have uploaded Supporting Information files, but you have not included a list of legends. Please add a full list of legends for your Supporting Information files after the references list.

Please review your reference list to ensure that it is complete and correct. If you have cited papers that have been retracted, please include the rationale for doing so in the manuscript text, or remove these references and replace them with relevant current references. Any changes to the reference list should be mentioned in the rebuttal letter that accompanies your revised manuscript. If you need to cite a retracted article, indicate the article’s retracted status in the References list and also include a citation and full reference for the retraction notice.

Response E.1: Thank you and the reviewers for the positive evaluation, specific feedback, and the opportunity to revise our manuscript for PLoS Global Public Health. We have responded to all comments below and highlighted all changes made to the manuscript.

We have included a list of legends for our supplemental material right after the references.

Furthermore, we have thoroughly reviewed our complete reference list, and confirm that this is complete and correct, with no papers that had been retracted. In response to R1’s comments, we have added the following references to our reference list:

References added:

1. Ogunwole SM, Golden SH. Social determinants of health and structural inequities—root causes of diabetes disparities. Diabetes Care. 2021;44(1):11-3.
2. Haw JS, Shah M, Turbow S, Egeolu M, Umpierrez G. Diabetes complications in racial and ethnic minority populations in the USA. Current Diabetes Reports. 2021;21:1-8.
3. Varga TV, Niss K, Estampador AC, Collin CB, Moseley PL. Association is not prediction: A landscape of confused reporting in diabetes–A systematic review. Diabetes Research and Clinical Practice. 2020;170:108497.
4. Valabhji J, Barron E, Bradley D, Bakhai C, Fagg J, O’Neill S, et al. Early outcomes from the English National health service diabetes prevention programme. Diabetes Care. 2020;43(1):152-60.
5. National Center for Chronic Disease Prevention and Health Promotion. Cost-Effectiveness of Diabetes Interventions. [https://www.cdc.gov/chronicdisease/programs-impact/pop/diabetes.htm]. Accessed: 26 Jan 2022.
6. Center for Disease Control and Prevention. National Center for Health Statistics. NHANES Survey Methods and Analytic Guidelines. [https://wwwn.cdc.gov/nchs/nhanes/analyticguidelines.aspx]. Accessed: 29 Mar 2023.
7. R Core Team. R: A language and environment for statistical computing. R Foundation for Statistical Computing, Vienna, Austria. 2013. http://www.R-project.org/
8. Kale MS, Korenstein D. Overdiagnosis in primary care: framing the problem and finding solutions. BMJ. 2018;362.

**Reviewer #1:**

R1.1: Nice idea and topic very well presented by authors. Here are my comments:
Introduction: More literature needs to be added to the intro explaining more details about the current prediction models, how they work, their impact and their relevance to NHANES data.

Response R1.1: Thank you for your positive evaluation of our paper. We have added additional details on prediction models, how they work, and their impact and relevance to the Introduction, as you suggested.

Introduction: *“Early detection of individuals who are at high risk for developing type 2 diabetes is a powerful strategy to tackle the diabetes epidemic, as it allows for targeted prevention.”*

Introduction: *“To the best of our knowledge, despite the availability of numerous other US-based prognostic risk prediction models and the success of prognostic models forecasting cardiovascular diseases, very few or none of them are currently implemented in routine clinical practice. Prognostic models for type 2 diabetes, incorporating information on demographic, socioeconomic, lifestyle, familial, and biological factors, can be used to calculate a “personalized” probability of developing the disease in a certain time window. The United Kingdom-based ‘Healthier You: National Health Services Diabetes Prevention Programme’, for example, has allowed the identification of thousands of individuals at high risk of developing type 2 diabetes, thereby enabling early intervention that successfully reduced their type 2 diabetes risk via structured support, adoption of healthier lifestyles, and reductions in adiposity and glycemia. Consequently, we do expect the implementation of prognostic type 2 diabetes prediction models in the US, as well as other countries, in the near future, largely due to financial incentives; prevention of type 2 diabetes and its comorbidities (e.g. obesity, and cardiovascular diseases) and complications will be more cost-effective in the long run than the treatment of them. Furthermore, the wide implementation and success of prognostic predictive models for cardiovascular diseases have already paved the way for how such models can be widely utilized to prevent disease.”*

Introduction: “*In this project, we set out to investigate whether the PRT and two key US-based prognostic prediction models for type 2 diabetes that informed the current guidelines, demonstrate consistent (mis)calibration across Black and White non-Hispanic US residents in the Continuous National Health and Nutrition Examination Survey (NHANES) survey.*”

R1.2: Introduction: More explanation about the impact of misdiagnosis of diabetes depending on race should be included showing recent references

Response R1.2: Thank you for pointing out this omission. We have included further details on this in the Introduction as suggested and referred to recent papers to back our statements (see references above in Response E.1 and the manuscript file).

Introduction: *“Such unequal predictions would exacerbate the systemic healthcare inequalities we are currently seeing, which stem from socioeconomic inequalities, differential health literacy and access to healthcare, and various forms of discrimination between majority and minority populations. For instance, biased type 2 diabetes prediction models that fail to identify at-risk individuals among racial minorities accurately would likely sustain or worsen the current disparities in developing diabetic complications. These complications include albuminuria, retinopathy, poor glycemic control, and cardiovascular diseases.”*

R1.3: Methodology: The process applied to NHANES data should be explained in more clarity mentioning the confounders and stratification of the sample in separate headings.

Response R1.3: Under the “Prediction models” heading, we clarified the components of the investigated risk prediction models - we refrain from using the term *confounders* here; while the original publications have likely used this term in establishing their regression models that informed the prediction models analyzed here, in our paper, these variables are referred to as *predictors* due to the analytic approach we are utilizing. Although the model-specific predictors have been already presented in detail in **Appendix Table 1**, we agree with you that this information is important enough to indicate in the main text, thus we added this. Of note, we also present all the utilized predictors in **Figure 1**.

Methods: *“The three investigated models included the following predictors:*

*1.	PRT: age, sex, BMI, family history of diabetes, history of hypertension, and physical activity*

*2.	Framingham Offspring Risk Score: sex, fasting glucose, BMI, HDL-C, family history of diabetes, triglycerides, systolic and diastolic blood pressure, anti-hypertensive medication use*

*3.	ARIC Risk Model: age, race, family history of diabetes, fasting glucose, systolic blood pressure, waist circumference, height, HDL-C, triglycerides.”*

Under the “Statistical analysis” heading, we added the following sections to clarify the stratification of the sample. We also clarified the weighting procedure by adding a reference.

Methods: *“For race-specific analysis, the cohort was stratified to non-Hispanic Whites (n=8,674), and non-Hispanic Blacks (n=1,313).”*

Methods: *“As an example, we used the overall and race-stratified NHANES population data from 1999-2000 to calculate 8-year average predicted incidence proportions using the Framingham Offspring Risk Score. We then compared these proportions to the estimates extracted from the US Diabetes Surveillance System database eight years after the study period (in this case, 2007).”*

Methods: *“Following the statistical guidelines by NHANES, these race- and year- specific averages were weighted to account for the survey design of the NHANES data”*

**Reviewer #2:**

R2.1: Overall, this study provides important insights into the potential racial bias in type 2 diabetes risk prediction models using NHANES data. The authors have followed a rigorous methodology and have presented their findings in a clear and accessible manner.

Response R2.1: Thank you for your positive evaluation of our paper.
